# Deciphering the Heterogeneous Microenvironment of Head and Neck Squamous Cell Carcinoma Through an Integrated Immune Inflammation Framework

**DOI:** 10.1155/ijog/8714244

**Published:** 2026-06-19

**Authors:** Jie Li, Shuxian Sun, Ziyu Ji, Jia Wang, Sichen Tang, Shuijie Shen, Xiaoya Shi

**Affiliations:** ^1^ Nantong University—Nantong Hospital of Traditional Chinese Medicine Clinical Innovation Research Center, Nantong Hospital of Traditional Chinese Medicine, Nantong, China, ntzyy.com; ^2^ Clinical Medical Research Center, Nantong Research Institute of Traditional Chinese Medicine, Nantong, China; ^3^ Nantong Hospital Affiliated to Nanjing University of Chinese Medicine, Nantong, China, bucm.edu.cn; ^4^ Clinical Innovation Research Center of Nantong University, Nantong Hospital Affiliated to Nanjing University of Chinese Medicine, Nantong, China, bucm.edu.cn

**Keywords:** head and neck squamous cell carcinoma, immune inflammation, machine learning, single-cell transcriptomics, tumor microenvironment

## Abstract

**Background:**

Head and neck squamous cell carcinoma (HNSC) exhibits substantial prognostic and microenvironmental heterogeneity. However, the integrated prognostic relevance of synergistic immune and inflammatory signatures in HNSC remains fully elucidated.

**Methods:**

Weighted gene coexpression network analysis (WGCNA) was integrated with curated immune‐ and inflammation‐related gene sets to identify key tumor‐associated candidate genes. A crucial phenotypic module exhibiting the strongest positive correlation with tumor status was prioritized, yielding six overlapping candidate genes. Utilizing the TCGA‐HNSC, GSE65858, and GSE41613 cohorts, we systematically compared multiple machine learning algorithms to construct a robust immune‐inflammation score (IIS), subsequently evaluating its prognostic efficacy and biological relevance.

**Results:**

The random survival forest model outperformed other algorithms and was utilized to establish the IIS. An elevated IIS was consistently predictive of inferior survival and served as an independent prognostic indicator. Furthermore, the IIS significantly correlated with specific immune infiltration patterns, immune checkpoint expressions, TIDE‐related features, tumor microenvironment scores, and distinct genomic mutation profiles, including tumor mutation burden. Notably, CSF2, IL1R2, and IL20RB were identified as pivotal model constituents, displaying cell type–specific and spatially discrete expression trajectories.

**Conclusions:**

The proposed IIS constitutes a robust, clinically relevant prognostic biomarker for HNSC, capturing the profound immune and genomic heterogeneity inherent in the disease.

## 1. Introduction

Head and neck squamous cell carcinoma (HNSC) is a highly heterogeneous malignancy that primarily arises from the mucosal epithelium of the oral cavity, pharynx, and larynx [1]. As a profound global health problem, HNSC exhibits high incidence and mortality, with clinical outcomes varying significantly across tumor sites, etiologies, and molecular subtypes [2]. Despite advances in surgery, radiotherapy, systemic therapy, and multimodal treatment, the prognosis of patients with advanced or aggressive HNSC remains unsatisfactory [3]. Therefore, conventional clinicopathological factors alone are insufficient to fully capture disease heterogeneity or provide accurate prognostic stratification.

Emerging studies highlight the critical role of the tumor microenvironment (TME) in HNSC progression [4, 5]. Immune dysregulation and chronic inflammation are closely associated with malignant transformation, tumor growth, invasion, metastasis, and treatment resistance [6]. Inflammatory signaling not only remodels the local microenvironment to facilitate immune evasion, but compromised antitumor immunity also actively fuels tumor evolution [7]. In HNSC, the highly complex cross‐talk between tumor cells and infiltrating immune or stromal cells manifests as profound interpatient variability regarding both long‐term prognosis and treatment responses.

Immunotherapy has improved outcomes in a subset of patients with recurrent or metastatic HNSC, and pembrolizumab‐based therapy has become an important first‐line treatment option [8–10]. However, only a proportion of patients achieve durable clinical benefit, suggesting that single biomarkers are insufficient to comprehensively reflect the immune status of HNSC [11]. Previous studies have investigated immune‐related or inflammation‐related signatures separately, but the integrated prognostic value of these two biological processes in HNSC remains unclear [12, 13]. A broader framework incorporating both immune and inflammatory features may improve prognostic assessment and provide deeper insight into disease biology.

To address this, we combined weighted gene coexpression network analysis (WGCNA) with predefined immune and inflammatory gene sets to identify tumor‐associated candidates in HNSC. We subsequently developed and validated a machine learning–driven immune‐inflammation model using the TCGA‐HNSC, GSE65858, and GSE41613 cohorts. Finally, the clinical relevance, immune landscape, genomic profiles, and single‐cell/spatial transcriptomic features linked to this score were thoroughly evaluated.

## 2. Methods

### 2.1. Data Collection and Preprocessing

Transcriptomic profiles and corresponding clinical annotations for patients with HNSC were retrieved from public repositories. The TCGA‐HNSC cohort was used as the training set, and the GSE65858 [14] and GSE41613 [15] cohorts were used as external validation sets for prognostic assessment. Prior to downstream analyses, expression matrices underwent rigorous standardization, and samples with incomplete survival data were excluded. For GEO datasets, when multiple probes mapped to a single gene symbol, their average expression value was calculated to represent the gene′s expression level.

### 2.2. Collection of Immune‐ and Inflammation‐Related Genes

Immune‐related genes were collected from the ImmPort Portal and InnateDB databases. Concurrently, inflammation‐related genes were derived from the Molecular Signatures Database (MSigDB), including HALLMARK_INFLAMMATORY_RESPONSE, HALLMARK_TNFA_SIGNALING_VIA_NFKB, HALLMARK_IL6_JAK_STAT3_SIGNALING, HALLMARK_COMPLEMENT, and GOBP_INFLAMMATORY_RESPONSE_TO_ANTIGENIC_STIMULUS. The complete lists of immune‐related and inflammation‐related genes are provided in Table S1. To extract candidate genes governing the tumor immune–inflammation axis, these curated gene lists were intersected with coexpression modules identified via WGCNA.

### 2.3. Identification of Tumor‐Associated Immune‐Inflammation Genes

To obtain robust tumor‐associated immune‐inflammation genes, genes from the tumor‐related WGCNA yellow module were intersected with the predefined immune‐related and inflammation‐related gene sets. Six overlapping genes were identified and retained as candidate genes for subsequent model construction.

### 2.4. Machine Learning Model Construction and Development of the Immune‐Inflammation Score (IIS)

To establish a resilient prognostic signature, we systematically integrated and evaluated multiple machine learning algorithms, including random survival forest (RSF), StepCox, Lasso, Ridge, Elastic Net, generalized boosted modeling (GBM), SuperPC, CoxBoost, and survival support vector machine. Candidate models were initially trained on the selected genes within the TCGA‐HNSC cohort and sequentially evaluated in the validation cohorts. Predictive performance was rigorously compared using the concordance index (C‐index); the algorithmic framework achieving the highest average C‐index across all three cohorts was selected as optimal. Based on this optimal algorithm, an IIS was computed for each individual patient. Patients were subsequently stratified into high‐ and low‐IIS risk groups based on the predefined threshold derived from the training cohort.

### 2.5. Survival and Prognostic Analyses

Overall survival (OS) discrepancies between the high‐ and low‐IIS groups were estimated via Kaplan–Meier (KM) survival analysis and quantified using the log‐rank test across both training and validation cohorts. Time‐dependent receiver operating characteristic (ROC) curves were constructed to determine the predictive accuracy of the IIS for 1‐, 3‐, and 5‐year survival outcomes. To evaluate whether the IIS constitutes an independent prognostic factor, univariate and multivariate Cox regression analyses were performed after adjusting for baseline clinicopathological variables, including tumor grade, clinical stage, and TNM classification. Decision curve analysis (DCA) was employed to benchmark the clinical utility of the IIS against conventional staging parameters. Furthermore, stratified subgroup survival analyses were conducted across clinical stages and TNM categories to assess the stability of the IIS.

### 2.6. Immune Infiltration and Immunotherapy‐Related Analyses

To characterize immune features associated with IIS, immune cell infiltration levels were estimated and compared between the high‐ and low‐IIS groups. The expression levels of representative immune checkpoint genes were also compared between the two groups. To explore the potential association between IIS and immunotherapy response, Tumor Immune Dysfunction and Exclusion‐related metrics, including dysfunction and exclusion scores, were analyzed, and predicted immunotherapy responses were compared between IIS groups. Immune‐related functional signatures were also evaluated. In addition, TME‐related scores, including TMEscore and ESTIMATE‐derived stromal and immune scores, were compared between groups.

### 2.7. Somatic Mutation and Tumor Mutation Burden (TMB) Analysis

Somatic mutation data from the TCGA‐HNSC cohort were analyzed to delineate genomic alteration patterns associated with the IIS subtypes. Somatic variants were characterized based on variant classification, variant type, single‐nucleotide variant (SNV) class, and top mutated gene frequencies. Landscape waterfall plots were generated to illustrate mutation patterns and altered cancer pathways across the high‐ and low‐IIS groups. TMB was calculated for each sample, and its relationship with the IIS was assessed via correlation analysis. Finally, prognostic cross‐stratification was performed based on TMB alone and in combination with the IIS.

### 2.8. Model Interpretation and Gene‐Level Prognostic Assessment

To quantify individual gene contributions to the prognostic signature, SHapley Additive exPlanation (SHAP) analysis was executed across the TCGA‐HNSC, GSE41613, and GSE65858 cohorts, with gene importance determined by mean absolute SHAP values. The prognostic performance of individual model genes was further substantiated across multiple clinical endpoints, including OS, disease‐specific survival (DSS), and progression‐free interval (PFI).

### 2.9. Single‐Cell and Spatial Transcriptomic Analyses

To further describe the cellular and spatial expression patterns of the model genes, single‐cell and spatial transcriptomic analyses were performed using public HNSC datasets. After quality control and normalization, dimension reduction and unsupervised clustering were used to identify major cell populations, including malignant cells, fibroblasts, endothelial cells, mono/macrophages, T‐cell subsets, B cells, plasma cells, mast cells, and myofibroblasts. The expression patterns of the model genes were then shown across different cell types. Spatial transcriptomic analysis was also used to examine the spatial distribution of major cell populations and model genes within tumors and to describe their spatial heterogeneity in the TME.

### 2.10. Statistical Analysis

All computational and statistical workflows were implemented within the R environment. For continuous variables, differences between two groups were evaluated using the two‐tailed Wilcoxon rank‐sum test. Survival trajectories were rendered using the KM method and assessed via the log‐rank test. Intervariable correlations were evaluated using Spearman′s rank correlation coefficient. For all statistical analyses, significance was defined as a two − tailed *p* value < 0.05.

## 3. Results

### 3.1. Identification of Immune‐ and Inflammation‐Related Candidate Genes in HNSC

**Figure 1:**
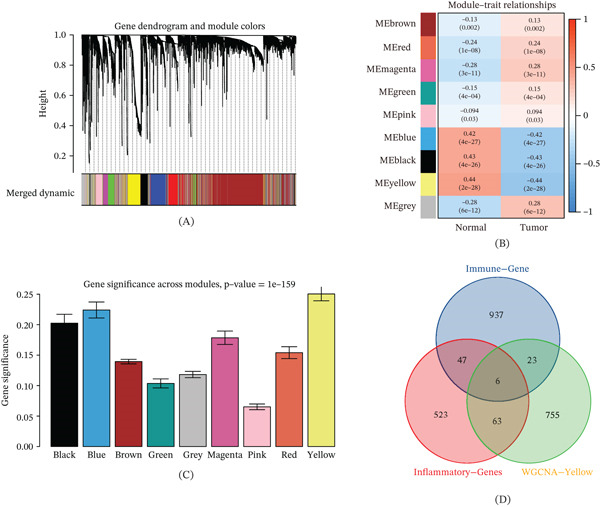
Screening of tumor‐associated immune‐ and inflammation‐related genes in HNSC. (A) WGCNA clustering dendrogram showing gene modules. (B) Module–trait relationships, showing that the yellow module was most positively associated with tumor status. (C) Gene significance across different modules. (D) Venn diagram showing the overlap among immune‐related genes, inflammation‐related genes, and WGCNA‐yellow module genes, identifying six candidate genes.

To identify immune‐ and inflammation‐related genes associated with tumor status in HNSC, we first performed WGCNA and classified genes into distinct coexpression modules. Following dynamic tree cutting and module merging, nine consensus modules were retained (Figure 1A). Module‐trait association analysis revealed that the yellow module exhibited the strongest correlation with the tumor phenotype and was consequently selected for further downstream exploration (Figure 1B). We subsequently evaluated gene significance (GS) across the modules, observing substantial variations in their trait relevancies (Figure 1C). Guided by these module–trait relationships, we focused on this key phenotypic module to capture core genes tightly linked to malignancy. To refine our candidate gene set, we performed an intersection analysis among the curated immune‐related genes, inflammation‐related genes, and the yellow module membership. This integrative analysis identified six overlapping genes, which were retained as core immune–inflammation‐related candidates for subsequent prognostic modeling (Figure 1D).

### 3.2. Development and Validation of the IIS in HNSC

To establish a robust prognostic signature for HNSC, we integrated multiple machine learning algorithms and systematically benchmarked their performance across the TCGA‐HNSC, GSE65858, and GSE41613 cohorts (Figure 2A,B). Among all candidate models, the RSF model achieved the highest average C‐index and was therefore selected to construct the IIS. Patients were stratified into high‐ and low‐IIS risk groups based on the optimal threshold. KM analysis showed that a high IIS was consistently associated with inferior prognosis in the TCGA‐HNSC cohort, and this finding was further validated in GSE65858 and GSE41613 (Figure 2C–E). Time‐dependent ROC analysis further supported the predictive value of IIS. In TCGA‐HNSC, the AUCs for 1‐, 3‐, and 5‐year survival were 0.935, 0.973, and 0.959, respectively, whereas those in GSE65858 were 0.712, 0.704, and 0.693, and those in GSE41613 were 0.701, 0.703, and 0.705 (Figure 2F–H). Univariate and multivariate Cox regression analyses demonstrated that IIS remained significantly associated with OS. After adjustment for clinicopathological variables, IIS remained an independent prognostic factor (Figure 2I,J). In addition, DCA showed that IIS provided a greater net benefit than conventional clinical variables for predicting 1‐, 3‐, and 5‐year survival (Figure 2K).

Figure 2Construction and validation of the immune‐inflammation score in HNSC. (A) Machine learning algorithms used for model construction. (B) Comparison of model performance across TCGA‐HNSC, GSE65858, and GSE41613 based on C‐index. (C–E) Kaplan–Meier (KM) survival curves of the high‐ and low‐IIS groups in TCGA‐HNSC, GSE65858, and GSE41613. (F–H) Time‐dependent ROC curves for 1‐, 3‐, and 5‐year survival in the three cohorts. (I, J) Univariate and multivariate Cox regression analyses of IIS and clinicopathological variables. (K) Decision curve analysis for 1‐, 3‐, and 5‐year survival prediction.

**Figure:**
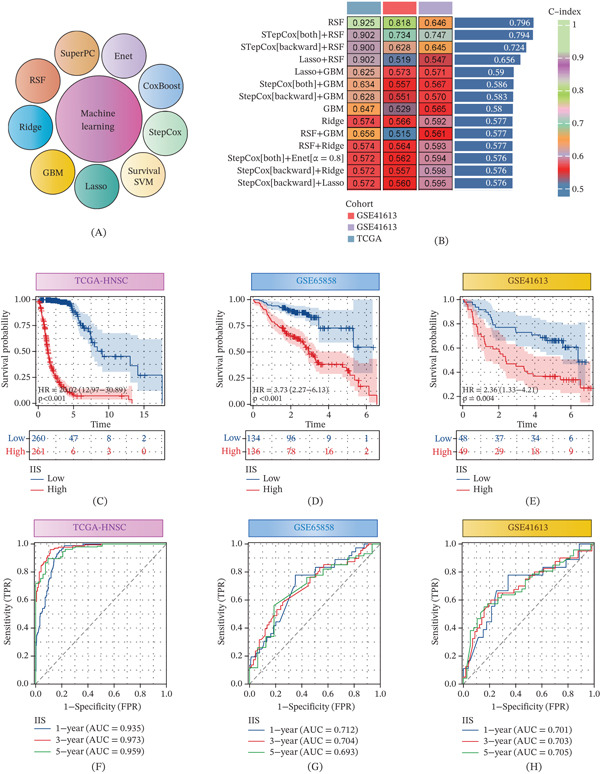
(A)

**Figure:**
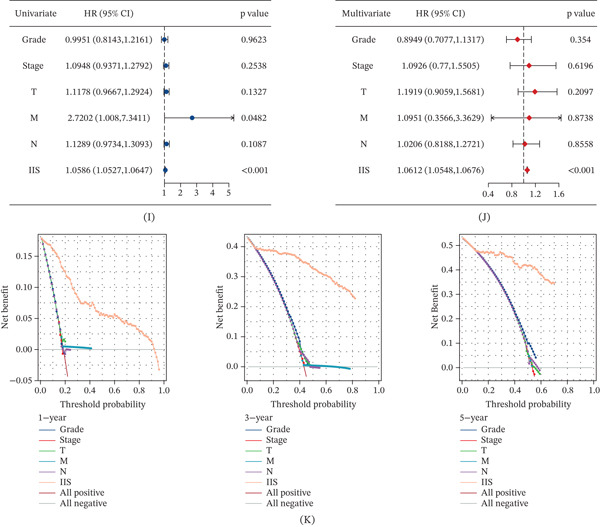
(B)

### 3.3. Subgroup Prognostic Value of the IIS in HNSC

**Figure 3:**
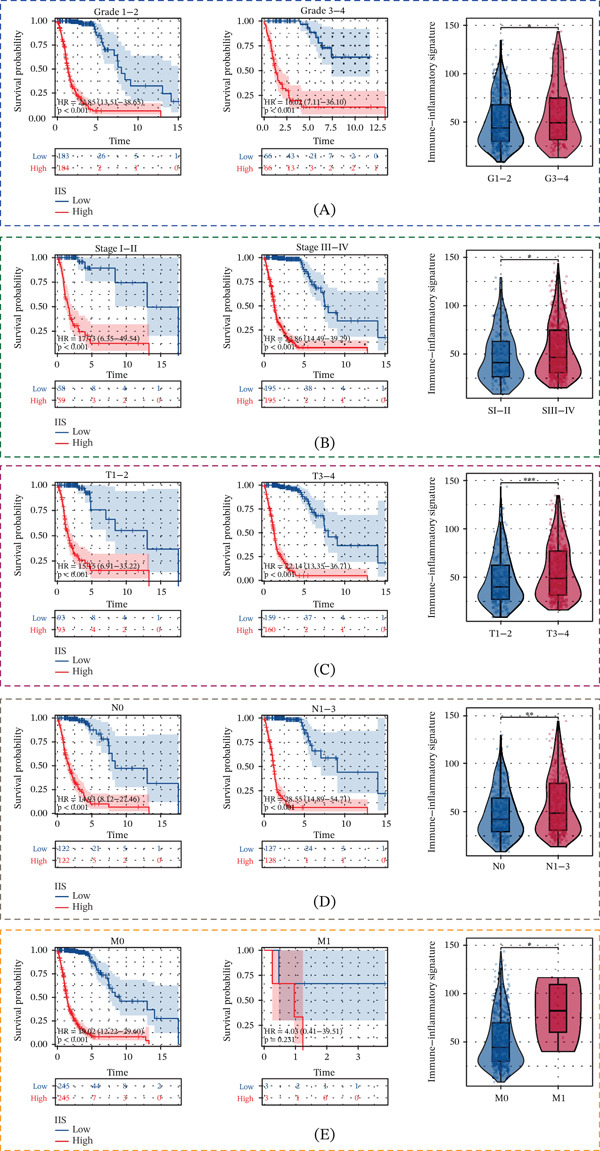
Subgroup prognostic analysis of the immune‐inflammation score in HNSC. (A–E) KM survival analyses and IIS distribution comparisons stratified by grade, clinical stage, T stage, M stage, and N stage, respectively. High IIS was associated with poorer overall survival across most clinicopathological subgroups and was enriched in patients with more advanced disease.

To further evaluate the stability and generalizability of the IIS, we performed stratified survival analyses across major clinical and pathological subgroups. A high IIS remained consistently predictive of compromised OS in patients with both low‐grade (G1–2) and high‐grade (G3–4) tumors (Figure 3A). Similar prognostic risk stratification was observed in early‐stage (Stages I–II) and advanced‐stage (Stages III–IV) diseases, with the high‐IIS group demonstrating significantly shorter survival trajectories in each stratum (Figure 3B). This robust performance extended across the primary tumor size categories, where patients with an elevated IIS experienced worse survival within both the T1–2 and T3–4 subsets (Figure 3C). In the distant metastasis stratum, the prognostic power of the IIS was maintained in the M0 subgroup, whereas no statistically significant difference was observed in the M1 subgroup (Figure 3D), which was likely due to the limited sample size of metastatic cases. Finally, the prognostic stratification of the score remained highly effective across nodal statuses, with high‐IIS predicting inferior survival in both node‐negative and node‐positive cohorts (Figure 3E).

### 3.4. Immune Landscape and Immunotherapeutic Relevance of the IIS in HNSC

We next examined immune features associated with IIS. Immune infiltration analysis revealed significant differences in several immune cell populations between the high‐ and low‐IIS groups, indicating marked heterogeneity in the tumor immune microenvironment (Figure 4A). Several immune checkpoint genes were also differentially expressed between the two groups, suggesting distinct immunoregulatory states across IIS subtypes (Figure 4B). We further evaluated the potential association between IIS and immunotherapy response. Predicted immunotherapy response differed significantly between the two groups, and IIS was associated with TIDE‐based metrics, supporting a potential link between IIS and immune escape (Figure 4C). Multiple immune‐related functional signatures also differed between the high‐ and low‐IIS groups, with the high‐IIS group exhibiting a more active immune‐related phenotype (Figure 4D). TME‐related scores, including TMEscore and ESTIMATE‐derived stromal and immune scores, were also significantly different between the two groups (Figure 4E,F).

Figure 4Immune landscape and immunotherapeutic relevance of the immune‐inflammation score in HNSC. (A) Comparison of immune cell infiltration between the low‐ and high‐IIS groups. (B) Differential expression of immune checkpoint–related genes between IIS subgroups. (C) Comparison of predicted immunotherapy response and analysis of TIDE‐related metrics between IIS subgroups. (D) Comparison of immune functional signatures between the two IIS groups. (E) Comparison of TME‐related scores between IIS subgroups. (F) Comparison of ESTIMATE, stromal, and immune scores between the low‐ and high‐IIS groups.

**Figure:**
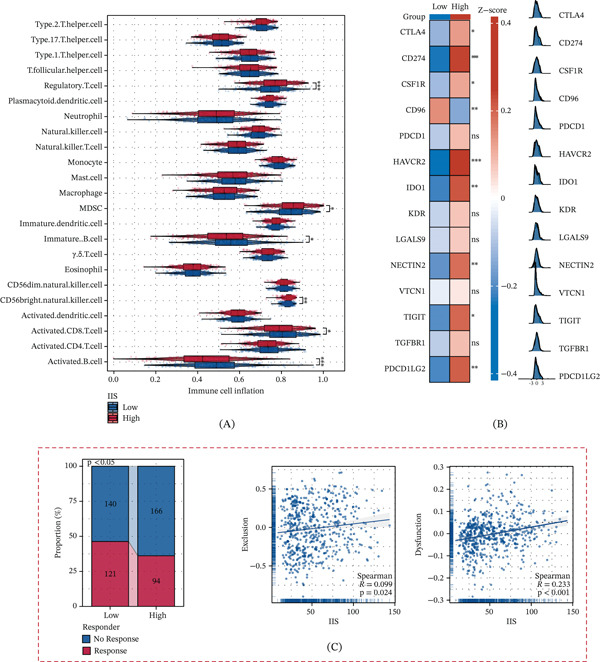
(A)

**Figure:**
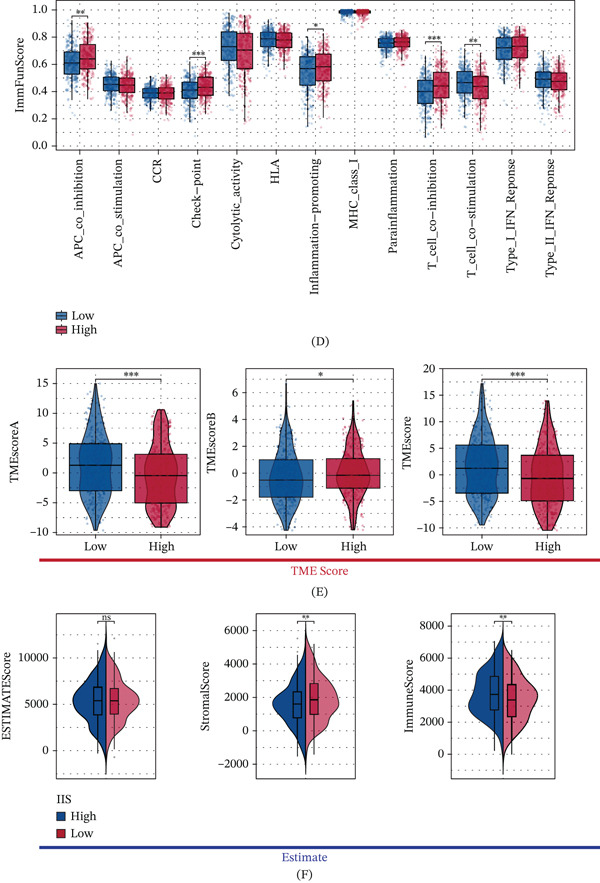
(B)

### 3.5. Genomic Alteration Landscapes Associated With the IIS in HNSC

To delineate the somatic mutational blueprints underlying the IIS subtypes, we investigated the genomic alteration profiles within the TCGA‐HNSC cohort. Overall, missense mutations represented the predominant variant classification, SNPs were the most common variant type, and C > T was the dominant SNV class. TP53 showed the highest mutation frequency (Figure 5A–C). Divergent mutational patterns were identified between the high‐ and low‐IIS groups. High‐resolution waterfall plots showcased notable discrepancies in the mutation frequencies of recurrently altered driver genes, as well as the somatic disruption of key oncogenic signaling pathways between the two risk strata (Figure 5D–G). Crucially, the high‐IIS group exhibited a significantly elevated TMB, and the IIS was positively correlated with TMB, albeit with a modest correlation coefficient (Figure 5H,I). Prognostic assessment revealed that a high TMB alone was predictive of abbreviated OS (Figure 5J). When integrating transcriptomic risk with genomic instability, cross‐stratification analysis demonstrated that patients in the IIS‐low/TMB‐low group achieved the most favorable clinical outcomes, whereas those in the IIS‐high/TMB‐low stratum experienced the most dismal prognosis (Figure 5K). These insights suggest that the IIS captures discrete genomic alteration signatures and offers complementary prognostic power when coupled with TMB.

Figure 5Genomic alteration landscape associated with the immune‐inflammation score in HNSC. (A–C) Overview of mutation classification, SNV class, and top mutated genes in HNSC. (D–G) Mutational landscape and pathway alteration profiles in the high‐ and low‐IIS groups. (H, I) Comparison of TMB between IIS subgroups and correlation between IIS and TMB. (J, K) Survival analyses based on TMB alone or in combination with IIS.

**Figure:**
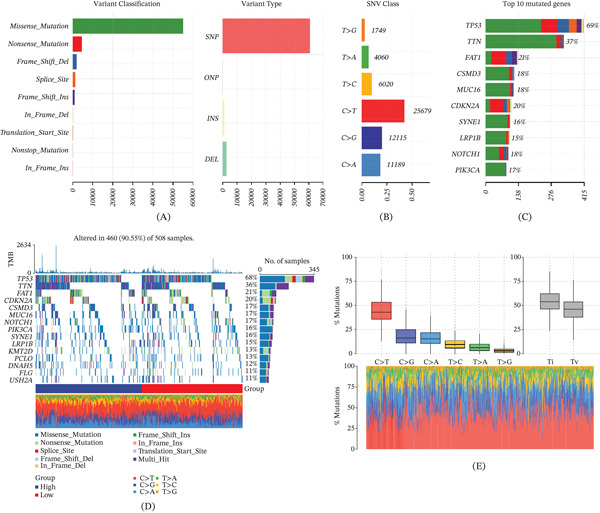
(A)

**Figure:**
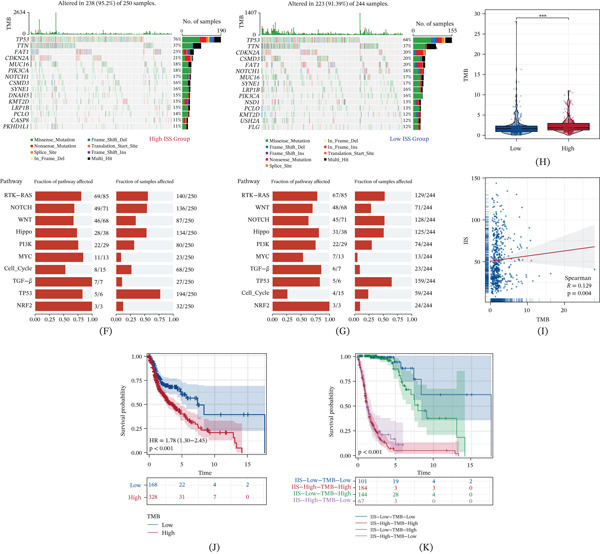
(B)

### 3.6. Interpretation and Prognostic Relevance of the Model Genes in HNSC

We next assessed the contribution of individual model genes using SHAP analysis in the TCGA‐HNSC, GSE41613, and GSE65858 cohorts. Across datasets, CSF2, IL1R2, and IL20RB were consistently identified as key contributors to model output, although their relative importance varied among cohorts, suggesting both cross‐cohort stability and cohort‐specific differences in gene effects (Figure 6A–C). Survival analyses further revealed distinct prognostic patterns for these genes. Higher CSF2 expression was associated with worse prognosis in TCGA‐HNSC, whereas IL20RB was associated with poorer OS and DSS. IL1R2 was significantly associated with PFI but showed more limited prognostic value for other survival outcomes (Figure 6D–F).

Figure 6Contribution and prognostic significance of key model genes in HNSC. (A–C) SHAP‐based interpretation of model genes in the TCGA‐HNSC, GSE41613, and GSE65858 cohorts. (D–F) Survival analyses of CSF2, IL1R2, and IL20RB across different clinical endpoints.

**Figure:**
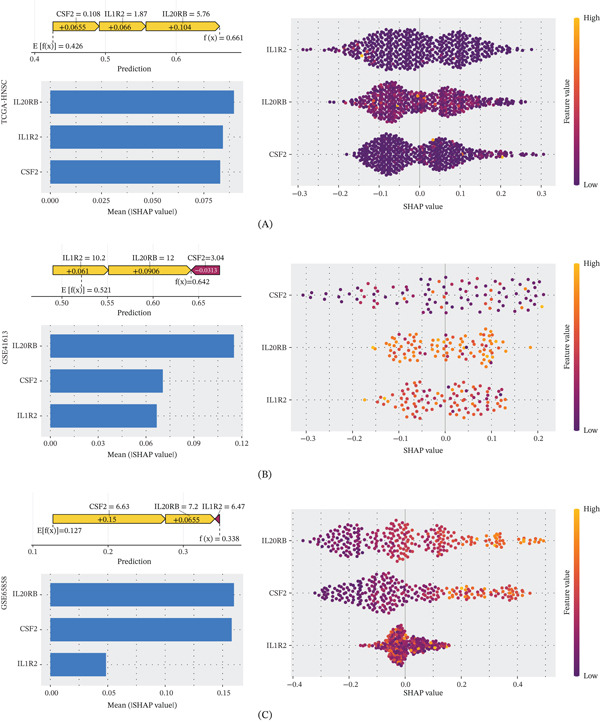
(A)

**Figure:**
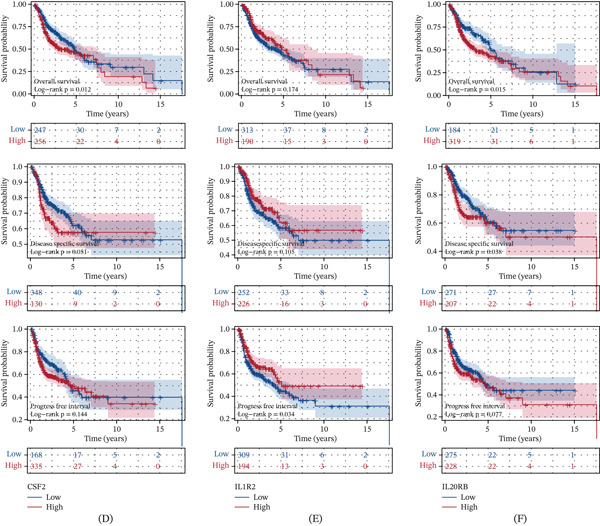
(B)

### 3.7. Single‐Cell and Spatial Transcriptomic Characterization of Model Genes in HNSC

To further characterize the cellular context of the model genes, we analyzed their expression in single‐cell and spatial transcriptomic datasets. Single‐cell analysis identified distinct cell clusters in HNSC, including malignant cells, fibroblasts, endothelial cells, mono/macrophages, T‐cell subsets, B cells, plasma cells, mast cells, and myofibroblasts (Figure 7A,B). Among the three model genes, IL20RB was predominantly enriched in malignant cells, whereas IL1R2 was mainly expressed in exhausted CD8+ T cells and mono/macrophage populations. By contrast, CSF2 showed generally low expression across cell subsets (Figure 7C–I). Spatial transcriptomic analysis further demonstrated marked intratumoral heterogeneity in cellular composition. Malignant cells and fibroblasts represented the major cell populations within tumor regions, whereas the spatial distributions of immune and stromal cells varied across locations (Figure 7J,K). Consistent with the single‐cell results, IL20RB showed relatively prominent spatial expression, IL1R2 displayed localized enrichment, and CSF2 remained weakly expressed (Figure 7L–O). These findings indicate that the model genes exhibit distinct cell‐type‐specific and spatial expression patterns in HNSC.

Figure 7Single‐cell and spatial transcriptomic characterization of model genes in HNSC. (A, B) Single‐cell clustering and cell‐type composition of HNSC samples. (C) Dot plot showing expression of marker genes across cell populations. (D–I) Single‐cell expression patterns of IL20RB, CSF2, and IL1R2. (J, K) Spatial distribution and composition of major cell types. (L–O) Spatial expression patterns of the three model genes.

**Figure:**
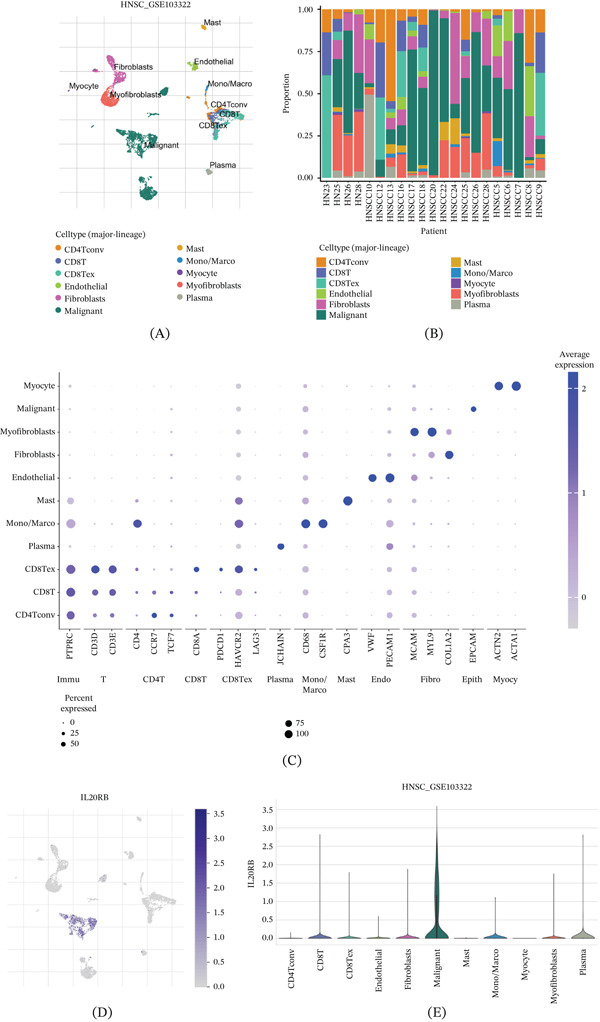
(A)

**Figure:**
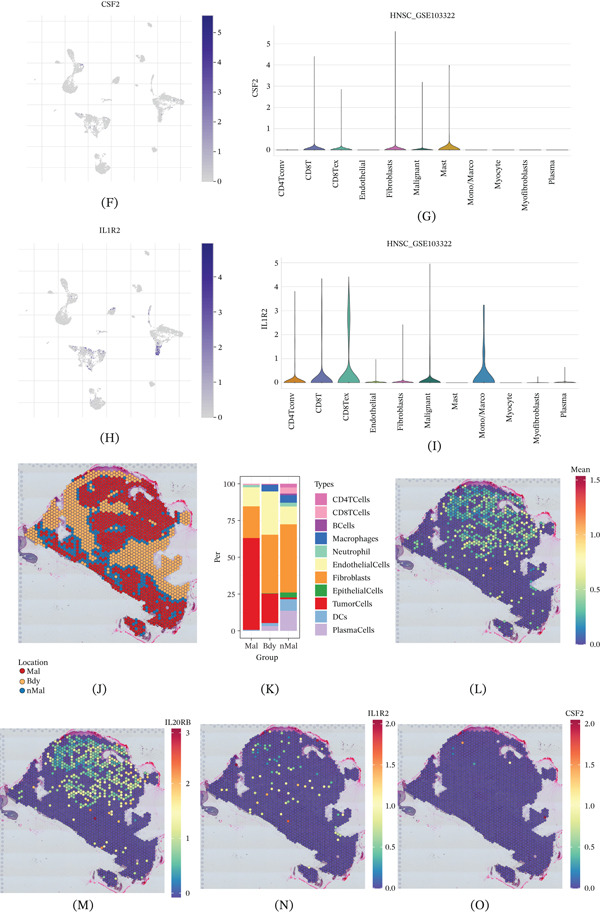
(B)

## 4. Discussion

In the present study, we developed and validated a novel immune‐inflammation prognostic model for HNSC by integrating tumor‐associated coexpression modules with curated immune‐ and inflammation‐related gene signatures, followed by machine learning–driven algorithmic optimization. The established IIS demonstrated robust and reproducible prognostic performance across the TCGA‐HNSC, GSE65858, and GSE41613 cohorts, remaining an independent prognostic indicator after rigorously adjusting for baseline clinicopathological variables and retaining its risk‐stratification efficacy across most clinical subgroups. These findings collectively suggest that the synchronized immune–inflammation axis effectively captures biologically meaningful heterogeneity in HNSC, offering clinical insights that extend beyond conventional pathological staging parameters.

The biological rationale underlying this model is supported by the established role of chronic inflammation in cancer development and tumor progression [16, 17]. Cancer‐related inflammation promotes tumor cell survival, angiogenesis, metastasis, and immune suppression and is now recognized as a hallmark of tumor evolution [18–20]. In HNSC, the TME is very complex, and interactions among tumor cells, immune cells, stromal cells, and inflammatory factors together influence disease behavior and treatment response [21, 22]. Our findings support the concept that integrating inflammatory and immune‐related signals may more effectively reflect tumor aggressiveness than either process alone.

An important finding of this study is that high IIS was associated not only with poor survival but also with pronounced immune heterogeneity. The high‐IIS group exhibited distinct immune infiltration patterns, differential expression of multiple immune checkpoint molecules, alterations in immune functional signatures, and significant differences in TME‐related scores. These findings suggest that high‐IIS tumors may exist in an immune‐active yet functionally dysregulated state characterized by concurrent inflammatory activation and immune suppression or exhaustion. This interpretation is biologically plausible in HNSC, where immune infiltration alone does not necessarily translate into effective antitumor immunity.

Our analyses related to immunotherapy also support the possible clinical value of IIS. Immune checkpoint blockade has become an important treatment option for recurrent or metastatic HNSC, and there is strong interest in biomarkers that can better show differences in treatment response [23, 24]. Clinical trials such as KEYNOTE‐048 established pembrolizumab‐based therapy as a standard first‐line treatment for selected patients, but long‐term benefit is still limited to only some cases [10]. In this setting, the strong association between the IIS and TIDE‐related metrics suggests that the score effectively reflects, at least in part, the status of immune evasion and potential responsiveness to ICB. Although these findings warrant further prospective validation in real‐world immunotherapy‐treated cohorts, they suggest that the IIS may function as a multicomponent biomarker bridging prognosis and therapeutic selection.

Furthermore, at the genomic level, we identified discrete mutational landscapes between the two IIS risk subgroups, with the IIS being positively associated with TMB. Notably, despite a modest correlation coefficient, integrating the transcriptomic‐derived IIS with genomic TMB significantly enhanced prognostic stratification. This synergy aligns with the known molecular heterogeneity of HNSC, where somatic driver alterations and continuous TME remodeling jointly dictate clinical outcomes.

Another strength of this study is the use of SHAP, single‐cell, and spatial transcriptomic analyses to improve biological interpretation. SHAP analysis identified CSF2, IL1R2, and IL20RB as major contributors to the model output, whereas single‐cell and spatial analyses showed different cell‐type and intratumoral localization patterns for these genes. This multilevel design moves the model beyond simple statistical prediction and provides an initial cellular context for understanding how the immune–inflammation axis may work in HNSC. Recent single‐cell and spatial studies have also shown that spatial organization and cell interactions are important factors of immune function and treatment vulnerability in head and neck cancer [25, 26].

Despite these insights, several limitations must be acknowledged. First, this investigation was retrospective in nature and relied exclusively on publicly available datasets, which may introduce inherent selection biases and platform‐associated batch effects. Second, although the model underwent independent external validation, large‐scale, prospective validation within multicenter clinical cohorts is still strictly required. Third, the immunotherapeutic responses inferred in this study were predominantly derived from computational surrogates rather than authentic clinical ICB datasets. Finally, the definitive mechanistic roles of the key model constituents—specifically their direct impact on immune remodeling and inflammatory cascades within HNSC—demand rigorous in vitro and in vivo experimental validation.

## 5. Conclusion

We developed a stable IIS for HNSC. This score can help classify patients by prognosis and reflects clear differences in the TME, genomic alterations, and cell type–specific gene expression patterns. These results show that combining immune and inflammatory features may be useful for biomarker development in HNSC and may also support future studies on risk assessment and treatment selection.

## Author Contributions

Jie Li, Shuxian Sun, Ziyu Ji, and Jia Wang contributed equally to this work. Jie Li and Xiaoya Shi conceived and designed the study, and were responsible for funding acquisition. Jie Li, Shuxian Sun, Ziyu Ji, and Jia Wang performed data collection, bioinformatics analyses, and model construction. Sichen Tang and Xiaoya Shi contributed to data interpretation and validation analyses. Shuijie Shen and Xiaoya Shi supervised the study and critically revised the manuscript. Jie Li drafted the manuscript. All authors reviewed and revised the final manuscript.

## Funding

This work was supported by the Nantong Social and People′s Livelihood Science and Technology Project (Grant No. MS2025052), and the Nantong University Special Research Fund for Clinical Medicine (Grant Nos. 2025JY048 and 2025LY072).

## Disclosure

All authors approved the final manuscript.

## Ethics Statement

The authors have nothing to report.

## Conflicts of Interest

The authors declare no conflicts of interest.

## Supporting information


**Supporting Information** Additional supporting information can be found online in the Supporting Information section. Table S1: The lists of immune‐related and inflammation‐related genes.

## Data Availability

All data supporting the findings of this study are included within the article and its Supporting Information. Additional datasets that are not explicitly presented are available from the corresponding authors upon reasonable request.
